# Obliterations of the Superior Vena Cava

**Published:** 1914-02

**Authors:** 


Obliterations of the Superior Vena Cava. Hebrard, *Gazette des Hôpitaux,* August 2, 1913. Obliteration of the superior vena cava has only recently been studied, the first reliable observations being made by Oulmont in 1856. This writer differentiated obstructions due to concretions within the vein from those due to external compression, and assigned a very gloomy prognosis to the conditions. Lately the part played by chronic inflammatory conditions of the mediastinum in bringing about obstruction has been demonstrated, and the outlook in certain cases is now known to be less serious than was formerly thought to be the case.

Impermeability of the superior vena cava may be due to malignant masses in the vein itself, to the thrombosis produced as a result of such masses, or to thrombosis indirectly produced in such cases by the consequent phlebitis. More commonly, however, the obstruction is due to external compression; compression due to the increased size of a neighboring organ, as in the case of an aortic aneurysm, tuberculosis of the tracheo-bronchial glands, malignant disease of an enlarged thyroid gland, to malignant disease of the mediastinum, or of the lung, and finally to chronic mediastinitis. Aneurysm of the aorta and chronic mediastinitis are the two most important causes to remember, in that the former is the most common, and it is among the latter group that the curable forms of obliteration are found. Chronic mediastinitis may be due to syphilis, tuberculosis, mediastinal pleurisy, mediastino-pericarditic inflammation, repeated infections of the respiratory tract, and rarely to acute rheumatism. Most important to realize that syphilis is a frequent factor in the production of mediastinitis that tends to compress the superior vena cava, a mediastinitis that may be cured by appropriate treatment.

From the point of view of morbid anatomy it is difficult to differentiate malignant disease of the vein itself from malignant disease of the adjacent organs which are generally affected at the time of the autopsy. Compression of the vein by an aortic aneurysm may or may not be complete.

As a rule the first effect of the aneurysm on the vein is to set up an adhesive inflammation between the opposing walls of the latter; if the compression goes no further, blood may still pass through the vein, between the interstices of these adhesions. In other cases where the compression has been severe, the trunk of the vein is found flattened upon the aneurysmal sac and its lumen completely obliterated.

Both fusiform and saccular aneurysms are capable of producing these consequences; in rare cases the vein has been invaginated by a small hemispherical dilatation of the aorta situated immediately above its origin from the heart. In some cases the vein has been found compressed between a dilatation of the aorta and an enlarged suppurating mediastinal gland.

Mediastinitis of syphilitic origin most commonly affects the antero-superior part of the mediastinum, and so readily compresses the vein. In certain types of this condition all the tissues of the mediastinum ultimately become matted together and constricted by the fibrosis that results, the fibrous tissue in such cases being exceptionally tough and creaking beneath the scalpel. The superior vena cava in such a case is found completely surrounded by the fibrous tissue; its lumen is diminished, and occupied by an organized clot. This clot may extend into the veins of the neck and upper extremities.

According to Renault the superior vena cava is one of the sites of election of tertiary syphilis. Tuberculous mediastinitis generally effects the lower parts of the mediastinum, but may in some cases compress the superior vena cava. The condition is usually found along with tuberculous tracheo-bronchial glands. Cases in which the vein is compressed as a result of a tuberculo-syphilic mediastinitis are occasionally met with in infants who have inherited syphilis. The mediastinitis of acute rheumatism is almost always associated with pericarditis and endocarditis. It should be remembered that syphilis, tuberculosis, and malignant disease, in addition to their direct effects on the vein, may also cause compression by enlarging the tracheo-bronchial glands.

The intra-thoracic tumors that tend to compress the superior vena cava are in the great majority of cases malignant, but cases have been recorded in which the pressure was due to benign growths, fibromata, lipomata, hydatid cysts, and dermoid cysts. A retrosternal prolongation of an enlarged thyroid gland has been known to produce the same result.

The results of compression of the superior vena cava are three: œdema of the upper part of the body, face, arms, chest and abdomen as far as the umbilicus: cyanosis of the face: and dilated veins over the upper part of the body. The author devotes some time to the discussion of the collateral circulation in these cases; he divides it into the cavo-caval system and the cavo-portal. The former consists of anastomoses between the internal and external mammary veins on the one hand, and the superficial abdominal veins on the other; of communications between the superior and inferior diaphragmatic veins; and between the azygos and intercostal veins and the posterior extracostal veins.

The porto-caval system consists of communications between the upper diaphragmatic vein and that of the suspensory ligament of the liver; the internal mammary and intercostal veins with the para-umbilical veins; the œsophageal veins with those of the cardia; and the azygos vein with the splenic through the intervention of the azygosplenic vein of Smieden.—Translated by Donald Core, for *Med. Chron.*

